# High-intensity mechanical therapy for loss of knee extension for worker's compensation and non-compensation patients

**DOI:** 10.1186/1758-2555-2-26

**Published:** 2010-10-12

**Authors:** Amanda L Dempsey, Thomas P Branch, Timothy Mills, Robert M Karsch

**Affiliations:** 1 Department of Orthopaedic Surgery and Sports Medicine, University of Kentucky College of Medicine, 138 Leader Ave., Lexington, KY 40506-9983, USA; 2 University Orthopaedics & Sports Medicine, 1014 Sycamore Dr., Suite B, Decatur, GA 30030, USA

## Abstract

**Background:**

Knee flexion contractures have been associated with increased pain and a reduced ability to perform activities of daily living. Contractures can be treated either surgically or conservatively, but these treatment options may not be as successful with worker's compensation patients. The purposes of retrospective review were to 1) determine the efficacy of using adjunctive high-intensity stretch (HIS) mechanical therapy to treat flexion contractures, and 2) compare the results between groups of worker's compensation and non-compensation patients.

**Methods:**

Fifty-six patients (19 women, 37 men, age = 51.5 ± 17.0 years) with flexion contractures were treated with HIS mechanical therapy as an adjunct to outpatient physical therapy. Mechanical therapy was only prescribed for those patients whose motion had reached a plateau when treated with physical therapy alone. Patients were asked to perform six, 10-minute bouts of end-range stretching per day with the ERMI Knee Extensionater^(r) ^(ERMI, Inc., Atlanta, GA). Passive knee extension was recorded during the postoperative visit that mechanical therapy was prescribed, 3 months after beginning mechanical therapy, and at the most recent follow-up. We used a mixed-model 2 × 3 ANOVA (group × time) to evaluate the change in passive knee extension between groups over time.

**Results:**

Regardless of group, the use of adjunctive HIS mechanical therapy resulted in passive knee extension deficits that significantly improved from 10.5° ± 5.2° at the initial visit to 2.6° ± 3.5° at the 3 month visit (p < 0.001). The degree of extension was maintained at the most recent follow-up (2.0° ± 2.9°), which was significantly greater than the initial visit (p < 0.001), but did not differ from the 3 month visit (p = 0.23). The gains in knee extension did not differ between worker's compensation and non-compensation patients (p = 0.56).

**Conclusions:**

We conclude that the adjunctive use of HIS mechanical therapy is an effective treatment option for patients with knee flexion contractures, regardless of whether the patient is being treated as part of a worker's compensation claim or not.

## Background

Flexion contractures are not uncommon following major knee surgery, and the loss of knee extension has been described as the most common complication after anterior cruciate ligament reconstruction (ACLR). Postoperative loss of knee extension, sometimes referred to as flexion contractures, has been reported in 8% to 25% of patients having undergone total knee arthroplasty (TKA) or ACLR [1-4]. TKA patients with losses of knee extension of less than 10° in the first 3 postoperative months generally regain motion over the course of the first two postoperative years, with only 8% demonstrating lasting residual motion restriction [5]. On the contrary, 58% of patients with more severe motion restriction (≥10°) were reported to have residual loss of knee extension two years after TKA.

By limiting a patient's ability to properly accept weight during gait, even one degree of missing knee extension may negatively affect clinical outcomes [6]. Losses of knee extension after TKA led to poorer outcomes related to pain, walking, stair-climbing, and function [7]. Patients with flexion contractures often walk with a bent-knee gait, thus increasing loading to the quadriceps and increasing contact forces in the patellofemoral joint [8]. Walking distance is often reduced for ACLR patients with postoperative loss of extension, as the disadvantaged position and increased demand during bent-knee gait may lead to either quadriceps weakness and/or an earlier onset of quadriceps fatigue [9]. An inability to achieve full extension at a mean follow-up of 14 years after ACLR has been reported to result in both significantly reduced subjective outcome scores and a significantly greater prevalence of osteoarthritis in the involved knee [10]. Furthermore, an inability to achieve full postoperative extension after TKA has been reported to lead to a more rapid degeneration of the contralateral knee [11].

Surgical procedures, such as arthroscopic lysis of adhesions, may successfully improve the range of knee extension [12]. While arthroscopic lysis of adhesions has been demonstrated to be an effective treatment for patients with persistent loss of knee extension[12], a recent study reported that a greater number of worker's compensation patients failed the procedure when compared to non-compensation patients [13]. Regardless of procedure, poorer postoperative outcomes have historically been reported for worker's compensation patients when compared to non-compensation patients. In a meta-analysis published in the Journal of the American Medical Association, Harris et al.[14] reported that 175 of 211 studies evaluated demonstrated worse outcomes for compensation patients, and that the effect was "significant, clinically important, and consistent." Specific to the knee, significantly worse outcomes have been reported for worker's compensation patients following partial medial menisectomy[15], ACLR[16], autologous chondrocyte implantation[17], unicompartmental knee arthroplasty[18], and TKA [19-21].

With the reduced success of surgical interventions for worker's compensation patients, a greater emphasis must be placed on conservative treatments. Conservative treatments including physical therapy and home exercise programs are often used to treat flexion contractures [4]. While generally successful, as many as 48% of patients treated with these protocols may still require surgical intervention [4]. Similarly, studies evaluating the treatment of motion loss associated with shoulder adhesive capsulitis indicate that worker's compensation claims and/or pending litigation were associated with the eventual need for motion restoring surgery [22].

Similar to outcomes following surgery, rehabilitation protocols for patients with range of motion limitations have also been reported to be less effective with worker's compensation patients [22]. Whether being treated for wrist or shoulder conditions, worker's compensation patients did not respond as well to physical therapy interventions as non-compensated patients [22-24]. Moreover, worker's compensation patients with idiopathic adhesive capsulitis that were treated with a stretching exercise program were at greater risk of failing treatment and requiring a manipulation under anaesthesia or capsular release in order to improve motion [22].

For patients that have failed standard conservative treatment for two or more months, treatment protocols including physical therapy with the adjunctive use of mechanical therapy devices has been demonstrated to effectively treat flexion contractures [25-27]. By being available to patients in their own homes, adjunctive use of home mechanical therapy devices allow patients with loss of knee extension to be treated daily to improve motion and potentially avoid the need for motion-restoring surgery [27]. Mechanical therapy devices apply an overpressure stretch for a period of time in order to create plastic deformation of connective tissues [28]. Applying too low of a force, or applying force for too short of a period of time results in an elastic deformation that does not correspond with a lasting change in range of motion [29,30].

The effectiveness of mechanical therapy can be improved by increasing the number of treatment sessions per day or week, the duration of time spent stretching during each session, or the intensity of the torque being delivered to the tissue during each session [31]. Mechanical therapy devices can be categorized as either low-intensity stretch (LIS) or high-intensity stretch (HIS) devices. LIS devices are generally spring-loaded or apply force through the use of gearing such as low-load prolonged stretch and static progressive stretch devices, and HIS devices apply force to the knee using either pneumatics or hydraulics. HIS devices apply forces to the joint that are more similar to those applied by physical therapists, whereas LIS devices applied forces more similar to common home exercises [30]. By applying greater force, HIS devices may then be able to achieve an effective result in a shorter amount of time, both in terms of the amount of time used per day and the number of weeks or months of use [27,30,32].

In a recent study of more than 60,000 patients with knee arthrofibrosis, HIS was reported to result in significantly lower post-treatment knee-attributable health care utilization and medical costs, as well as a significantly reduced prevalence of post-treatment hospitalization when compared to patients treated with LIS devices [33]. It appears that the combination of force application that more closely replicates that of a physical therapist and the reduced treatment durations may have a beneficial effect on both patient compliance and outcomes, regardless of whether a patient was a worker's compensation case or not. The purposes of retrospective review were to 1) determine the efficacy of using adjunctive HIS mechanical therapy to treat flexion contractures, and 2) compare the results between groups of worker's compensation and non-compensation patients.

## Methods

As part of this IRB-approved retrospective protocol, we reviewed the medical records for all patients with flexion contractures that were treated with HIS mechanical therapy in addition to outpatient physical therapy. The 56 patients included in the study were treated by one of two study surgeons. Patient information including sex, age, and the diagnosis or surgical procedure that immediately preceded the prescription of mechanical therapy was recorded.(Additional File 1, Table S1) In addition, we recorded whether or not each patient was being treated as part of a worker's compensation claim.

**Figure 1:**
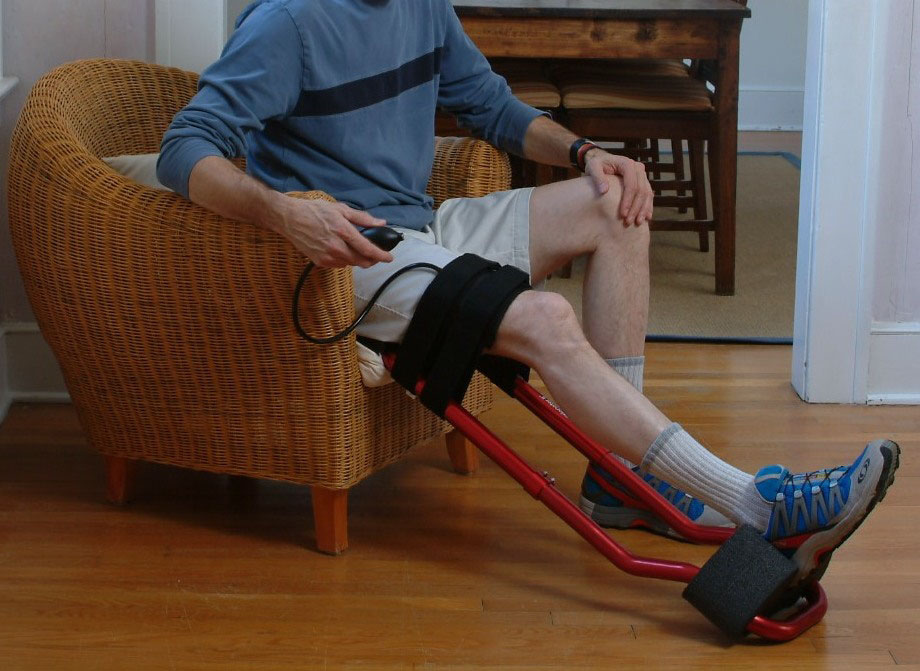
**High-intensity stretch mechanical therapy device used in this study**. The ERMI Knee Extensionater^(r) ^was used as an adjunct to physical therapy for patients with knee flexion contractures.

Mechanical therapy was only prescribed for those patients whose motion had reached a plateau when treated with physical therapy alone. As an adjunct to outpatient physical therapy, patients were asked to perform six 10-minute bouts of end-range stretching per day with the ERMI Knee Extensionater^(r) ^per the manufacturer's instructions for use (ERMI, Inc., Atlanta, GA; Figure 1). The ERMI Knee Extensionater^(r) ^is a device that increases passive knee extension through the use of a three-point bending system. A rigid frame is placed posterior to the patient's lower extremity, with the patient's heel placed in a contoured foam pad that discourages hip external rotation. An inflatable air cuff is then secured on the anterior aspect of the leg, proximal to the level of the patella. As the patient inflates the air cuff, the knee is moved to the end range of motion. Patients were instructed to inflate the air cuff until the point to where they feel they are recreating the intensity of the stretch that was provided by their physical therapist.

Passive knee extension was recorded during the office visit that mechanical therapy was prescribed, 3 months after beginning mechanical therapy, and at the most recent follow-up. The measurements were made by one of two investigators (RMK, TPB) using standard goniometry. Patients were measured in a supine position without the use of a second evaluator to apply overpressure. We used a mixed-model 2 × 3 ANOVA (Group × Time) to evaluate the change in passive knee extension over time, with Bonferroni post hoc analyses being used to determine the location of significant differences. Statistics 17.0 (SPSS, Inc., Chicago, IL) was used to perform all analyses, and an α-level of p ≤ 0.05 was considered significant.

## Results

**Figure 2:**
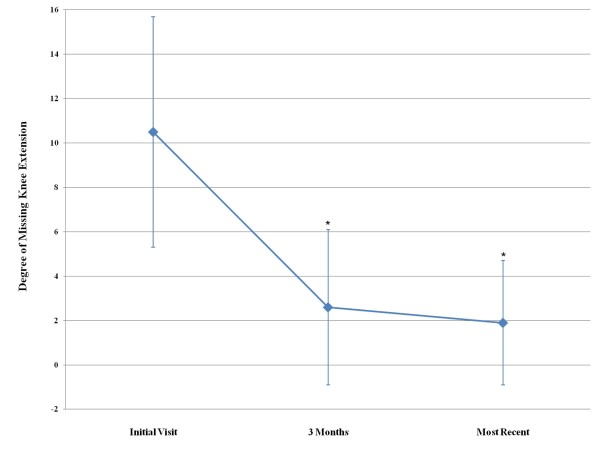
**Improvement of knee extension over time**. The degree of knee extension significantly improved from the initial visit and 3-month follow-up (p < 0.001), and was maintained between the 3-month and most recent follow-ups (p = 0.23). * indicates significantly different (p < 0.05) from initial visit

The mean follow-up for the sample was 13.7 ± 11.5 months (mean ± standard deviation). Regardless of group, the use of adjunctive HIS mechanical therapy resulted in passive knee extension deficits that significantly improved from 10.5° ± 5.2° at the initial visit to 2.6° ± 3.5° at the 3 month visit (p < 0.001). The degree of extension was maintained at the most recent follow-up (2.0° ± 2.9°), which was significantly greater than the initial visit (p < 0.001), but did not differ from the 3 month visit (p = 0.23).(Figure 2) The gains in knee extension did not differ between worker's compensation and non-compensation patients (p = 0.56, Table 1).

**Table 1:** Passive knee extension (degrees ± standard deviation) for worker's compensation and non-compensation patients at the initial visit, and 3-month and most recent follow-up visits

	Initial Visit	3-months	Most Recent Follow-up
Worker's Compensation Patients	11.1 ± 5.7	2.9 ± 4.3	2.4 ± 3.9
Non-Compensation Patients	10.3 ± 5.1	2.5 ± 3.2	1.9 ± 2.4

The majority of patients in this study underwent either knee arthroplasty or ACLR procedures. Both the 24 patients that underwent UKA or TKA and the 17 patients that underwent ACLR demonstrated the same progression seen in the overall results. Significant gains were seen between the pre- and post-treatment measurements, but no statistical differences were noted between the post-treatment measurements and those taken at the patients' most recent follow-up. There were no statistical differences between patients undergoing either arthroplasty or ACLR (p = 0.28). Passive extension for the UKA and TKA patients improved from 9.6° ± 4.2° at the baseline visit to 2.7° ± 2.8° at the 3 month visit and 2.2° ± 2.7° at the most recent follow-up. Passive extension for the ACLR patients improved from 10.8° ± 5.2° at the baseline visit to 0.8° ± 1.6° at the 3 month visit and 0.4° ± 1.1° at the most recent follow-up.

Of the two osteoarthritis patients treated with the HIS device, one demonstrated apparently long lasting improvements passive knee extension (10° improvement at 21 months). The other OA patient began HIS treatment with a 6° flexion contracture and did not demonstrate any improvement in passive knee extension (0° change). There were three other patients that demonstrated less than 2° of improved extension. A female patient with a 5° contracture began using the device 11 weeks status post primary TKA and did not demonstrate any improvement after four weeks of use. A male patient with a 5° contracture began using the device six weeks status post autologous hamstring double-bundle ACLR demonstrated 1° of improvement after nine weeks of use. Lastly, a male patient with a 4° contracture began using the device 14 weeks after a tibial plateau fracture and did not demonstrate any improvement after 11 weeks of use. Of the four patients that did not improve with the device, one patient was being treated as part of a worker's compensation claim and three were non-compensation patients.

## Discussion

The purposes of retrospective review were to 1) determine the efficacy of using adjunctive HIS mechanical therapy to treat flexion contractures, and 2) compare the results between groups of worker's compensation and non-compensation patients.

Patients with knee flexion contractures treated with the HIS device demonstrated a significant, 8-degree increase in passive knee extension, and these gains were maintained at the patients' most recent follow-up. Patients were asked to complete six, 10-minute sessions per day with the HIS device, which is 3 to 12 times lower than what has been previously reported protocols using a low-load, prolonged stretch device to treat knee flexion contractures. One such protocol asked patients to use the device three hours per day at a moderate intensity setting, and the patients treated with this protocol did not demonstrate significant improvement [32]. However, a small case series of four patients treated with the same low-load device asked patients to use the device at a higher intensity setting for 8 to 12 hours per day, and reported significantly improved knee extension [34]. Variability in the intensity, duration, and frequency of use with dynamic splints may potentially explain the mixed results that have been reported when treating flexion contractures [35].

The effectiveness of mechanical therapy can be improved by increasing the number of treatment sessions per day or week, the duration of time spent stretching during each session, or the intensity of the torque being delivered to the tissue during each session [31]. The product of multiplying the intensity, frequency, and duration of a treatment has been termed the Total End Range Time [31]. Previous authors have questioned whether there is an ideal Total End Range Time necessary to effectively treat flexion contractures [30]. In addition, previous authors have not only questioned whether there is an ideal total end range time, but also whether there is a minimum level of torque necessary to treat flexion contractures. It has been reported that 9.0 N-m of torque is necessary to maintain full extension in 18 patients with flexion contractures [32]. The high-intensity device used in the current study has been demonstrated to be able to apply 53.0 N-m of torque[30]; well above the minimum treatment threshold of 9.0 N-m. The combination of higher torque allows shorter treatment times and/or reduced treatment duration, and when the high-intensity device in the current study was used as an adjunct to physical therapy, it appeared to achieve a therapeutic relationship between the torque applied to the joint and the amount of daily use. We would like to stress that the patients in this study used the device as an adjunct to physical therapy, and not as a stand-alone treatment. As such, patients were able to supplement the passive range of motion gains made at home with the HIS device with supervised exercises in the outpatient physical therapy setting to improve quadriceps strength and activation in order to improve active as well as passive range of motion.

The ability of the treatment protocol used in the current study demonstrated similar results between worker's compensation and non-compensation patients. With the consistent reports of worse outcomes and increased risk of reoperation following either surgery or rehabilitation for worker's compensation patients, the lack of difference in improved knee extension in the current study between compensation and non-compensation patients is noteworthy. Because of the retrospective design of the current study, we can only speculate as to why HIS mechanical therapy significantly improved knee extension with both worker's compensation and non-compensation patients. It has been previously reported that shorter, more time efficient bouts of stretching may improve patient compliance and the efficacy of a treatment protocol[36], and we suspect that the reduced amount of daily use, as well as the overall shorter treatment durations associated with HIS mechanical therapy may have improved patient compliance, thus leading to more consistent range of motion improvements. Not only was motion improved with the use of adjunctive HIS mechanical therapy, the improvements were maintained at the most recent follow-up.

This study was not without limitation. While we were able to detect a significant treatment effect with the adjunctive use of the HIS device, we were unable to determine if gains in passive knee extension corresponded with improved clinical or functional outcomes as part of this retrospective study. In addition, the retrospective nature of this study limits our ability to control variability in the passive range of motion measurement techniques that were used. Range of motion measurements were made by two investigators, and we cannot be sure of the intra-rater reliability of these previously recorded measurements. During the time that study patients were treated, neither passive extension of the contralateral knee nor the amount of overpressure applied during the measurements was routinely recorded as part of the patient's medical records. Future prospective studies are thus warranted.

## Conclusions

We conclude that the adjunctive use of HIS mechanical therapy is an effective treatment option for patients with knee flexion contractures, regardless of whether the patient is being treated as part of a worker's compensation claim or not.

## Competing interests

Funding for this study was provided by ERMI, Inc. One author is a stockholder for ERMI, Inc., and holds a patent related to the device used in this study.

## Consent

Written informed consent was obtained from the patient for publication of the image. A copy of the written consent is available for review by the Editor-in-Chief of this journal.

## Authors' contributions

AD participated in the study design and coordination, data collection and analysis, and prepared the manuscript. TB, TM, and RK participated in the design of the study, data collection, interpretation of results, and helped draft the manuscript. All authors read and approved the final manuscript.

## Supplementary Material

Additional file 1 **Table S1. Patient demographics and diagnoses or surgeries that preceded the development of a knee flexion contracture**. The following acronyms are used in the table: DB ACLR = autologous hamstring double-bundle ACLR, SB ACLR = autologous hamstring single-bundle ALCR, HTO = high tibial osteotomy, LM = lateral menisectomy, LMR = lateral meniscus repair, LMT = lateral meniscus transplant, MCLR = Open medial collateral repair MM = medial menisectomy, MMT = medial meniscus transplant, MUA = manipulation under anesthesia, OA = osteoarthritis, ORIF = open reduction internal fixation, FB TKA = fixed-bearing PCL-sacrificing TKA, RP TKA = rotating platform total knee arthroplasty, UKA = fixed bearing unicompartmental arthroplasty Click here for file
